# Permissive effect of GSK3β on profibrogenic plasticity of renal tubular cells in progressive chronic kidney disease

**DOI:** 10.1038/s41419-021-03709-5

**Published:** 2021-04-30

**Authors:** Bohan Chen, Pei Wang, Xianhui Liang, Chunming Jiang, Yan Ge, Lance D. Dworkin, Rujun Gong

**Affiliations:** 1 Division of Kidney Disease and Hypertension, Department of Medicine, Rhode Island Hospital, Brown University School of Medicine, Providence, RI 02903 USA; 2 Division of Nephrology, Department of Medicine, University of Toledo College of Medicine, Toledo, OH 43614 USA

**Keywords:** Chronic kidney disease, Translational research

## Abstract

Renal tubular epithelial cells (TECs) play a key role in renal fibrogenesis. After persistent injuries that are beyond self-healing capacity, TECs will dedifferentiate, undergo growth arrest, convert to profibrogenic phenotypes, and resort to maladaptive plasticity that ultimately results in renal fibrosis. Evidence suggests that glycogen synthase kinase (GSK) 3β is centrally implicated in kidney injury. However, its role in renal fibrogenesis is obscure. Analysis of publicly available kidney transcriptome database demonstrated that patients with progressive chronic kidney disease (CKD) exhibited GSK3β overexpression in renal tubulointerstitium, in which the predefined hallmark gene sets implicated in fibrogenesis were remarkably enriched. In vitro, TGF-β1 treatment augmented GSK3β expression in TECs, concomitant with dedifferentiation, cell cycle arrest at G2/M phase, excessive accumulation of extracellular matrix, and overproduction of profibrotic cytokines like PAI-1 and CTGF. All these profibrogenic phenotypes were largely abrogated by GSK3β inhibitors or by ectopic expression of a dominant-negative mutant of GSK3β but reinforced in cells expressing the constitutively active mutant of GSK3β. Mechanistically, GSK3β suppressed, whereas inhibiting GSK3β facilitated, the activity of cAMP response element-binding protein (CREB), which competes for CREB-binding protein, a transcriptional coactivator essential for TGF-β1/Smad signaling pathway to drive TECs profibrogenic plasticity. In vivo, in mice with folic acid-induced progressive CKD, targeting of GSK3β in renal tubules via genetic ablation or by microdose lithium mitigated the profibrogenic plasticity of TEC, concomitant with attenuated interstitial fibrosis and tubular atrophy. Collectively, GSK3β is likely a pragmatic therapeutic target for averting profibrogenic plasticity of TECs and improving renal fibrosis.

## Introduction

Regardless of the original etiology, kidney fibrosis, characterized by renal tubular atrophy and excessive accumulation of extracellular matrix (ECM) in tubulointerstitium, is the hallmark of progressive chronic kidney disease (CKD), the final common pathway to end-stage renal failure, and the best predictor of renal survival^1^. Recently, a plethora of evidence indicates that renal tubular epithelial cells (TECs) are centrally implicated in the development and progression of renal fibrosis^2,3^. In response to various types of injuries, TECs undergo self-repair and adaptation to attain a new homeostatic equilibrium that would be compatible with their viability in the new environment. However, if the severity, frequency, or duration of the injury is beyond the repair capacity of TECs, TEC profibrogenic plasticity may arise, marked by cell dedifferentiation with distinctive features of loss of epithelial phenotypes and acquisition of mesenchymal characteristics. In addition, dedifferentiated TECs will release excess amounts of profibrotic cytokines such as connective tissue growth factor (CTGF) and plasminogen activator inhibitor-1 (PAI-1), which in turn act on neighboring TECs or myofibroblasts in a paracrine mode, eventually leading to ECM overproduction and kidney scarring^4,5^. Moreover, severely or chronically injured TECs may undergo cell cycle arrest at the G2/M phase and hence lose the ability to proliferate and repopulate the damaged tubules. Collectively, all these maladaptive plastic changes of TECs will act synthetically to cause kidney fibrosis but hinder the recovery of kidney function.

Glycogen synthase kinase (GSK) 3β is a ubiquitously expressed serine/threonine-protein kinase that acts as an integration point for multiple cellular pathways involved in glycogen biosynthesis, inflammation, mitochondrial dysfunction, and apoptosis^6^. Emerging data suggest that GSK3β plays an instrumental role in kidney injury. In experimental glomerular diseases, podocyte-specific knockout of GSK3β conferred a beneficial effect on podocyte injury and glomerular damage^7,8^. In murine models of acute tubular injury caused by a variety of nephrotoxic insults like acute renal ischemia-reperfusion, diclofenac, or paraquat, the renal tubular activity of GSK3β was evidently increased, and inhibition of GSK3β improved renal injury by ameliorating tubular cell apoptosis and damage^9–11^. However, it remains obscure whether GSK3β is involved in renal fibrogenesis in progressive CKD. Given the primacy of TEC maladaptive plasticity in the development and progression of kidney fibrosis, this study explored the role of GSK3β in TEC profibrogenic plasticity and kidney fibrosis in vitro in cultured TECs treated by transforming growth factor (TGF)-β1, and in vivo in the murine model of folic acid (FA) nephropathy.

## Materials and methods

### Cell culture and transient transfection

Conditionally immortalized murine proximal tubular epithelial cells (TKPT) were used as previously described^9^ and cultured at 37°C in DMEM/F12 supplemented with 5% Fetal Bovine Serum (FBS) in a humidified incubator with 5% CO_2_. TKPT have been authenticated and tested for mycoplasma contamination. Cells were seeded onto 60 mm Petri dishes. When reaching 50% confluency, cells were changed to serum-free DMEM/F12 for 12 h. Thereafter, cells were stimulated with different doses of TGF-β1 (0.5, 1, 2, or 4 ng/ml; R&D, Minneapolis, MN, USA) for 12 h, 24 h, or 48 h. Alternatively, cells were pretreated with different doses of TDZD-8 (2, 5, 10 μmol/L; Sigma-Aldrich, St. Loius, MO, USA), lithium chloride (LiCl, 2, 5, 10 mmol/L; Sigma-Aldrich) or forskolin (20 μmol/L, Cayman, Ann Arbor, MI, USA) for 30 min, and thereafter treated with TGF-β1 (2 ng/ml) for the indicated time. The eukaryotic expression vectors encoding the haemagglutinin (HA)-conjugated dominant-negative kinase-dead (KD), or constitutively active (S9A) mutant of GSK3β were transfected to TKPT as previously described^9^ by using Lipofectamine 2000 according to the manufacturer’s instructions (Invitrogen, Carlsbad, CA, USA). After transfection, cells were subsequently subjected to the indicated treatment.

### Animal study

#### Murine model of FA-induced CKD

Renal tubule-specific GSK3β knockout (KO) mice (C57BL/6 strain) and control littermates were generated by mating mice carrying the floxed GSK3β transgene with γ-glutamyltranspeptidase (γGT). Cre transgenic mice as previously elaborated^12^. Male KO or control mice aged 10 weeks were randomized (not blinded) to the following four treatment groups of six animals per group: (1) vehicle group: control mice only received vehicle treatment (0.3 M sodium bicarbonate) as a single intraperitoneal (*ip*.) injection. (2) control + FA group: control mice received a single dose of FA (*ip*., 250 mg/kg, Sigma-Aldrich) dissolved in 0.3 M sodium bicarbonate and sodium chloride (subcutaneous injection, *sc*., 1 mEq/kg) on day 7 after FA injury. (3) KO + FA group: KO mice received a single dose of FA (*ip*., 250 mg/kg). (4) control + FA + LiCl group: control mice received a single dose of FA (*ip*., 250 mg/kg) and then were treated with LiCl (*sc*., 40 mg/kg) on day 7. All mice were euthanized on day 14 after FA injection, followed by the collection of organs and serum for further investigation.

#### PCR genotyping for transgenic mice

A routine PCR protocol was used for genotyping tail DNA samples with the following primer pairs: for γGT.Cre genotyping, forward: 5’-AGGTGTAGAGAAGGCACTTAGC-3’ and reverse: 5’-CTAATCGCCATCTTCCAGCAGG-3’, which generated a 411-bp fragment; and for GSK3β genotyping, forward: 5’-GGGGCAACCTTAATTTCATT-3’ and reverse: 5’-GTGTCTGTATAACTGACTTCCTGTGGC-3’, which yielded 685-bp and 585-bp bands, respectively, for the floxed and wild-type alleles.

#### Serum blood urea nitrogen (BUN) measurements

Serum BUN levels were measured using a commercial assay kit (BioAssay Systems, Hayward, CA, USA) according to the manufacturer’s instruction.

#### Immunohistochemistry staining

Formalin‐fixed mouse kidneys were embedded in paraffin and 3-µm-thick sections were prepared. Sections were processed for immunohistochemistry staining. Immunoperoxidase staining was performed with a Vectastain ABC kit (Vector Laboratories, Burlingame, CA, USA) by using primary antibodies against GSK3β (12456, Cell Signaling Technology, MA, USA), Collagen I (sc-293182, Santa Cruz Biotechnology, CA, USA), PAI-1 (sc-8979, Santa Cruz Biotechnology), phosphorylated Histone H3 at serine 10 (pH3, 9701, Cell Signaling Technology) and phosphorylated cAMP response element-binding protein (CREB) at serine 133 (p-CREB, sc-81486, Santa Cruz Biotechnology). The immunoreactivity was assessed in a blind manner. The sections were visualized by using EVOS XL Core Imaging System (Thermo Fisher Scientific, Waltham, MA, USA).

#### Immunofluorescence staining

Cultured cells or cryosections of kidney samples were fixed with 4% paraformaldehyde (Sigma-Aldrich), permeabilized, and stained with primary antibodies against zonula occludens (ZO)-1 (617300, Invitrogen, MD, USA), E-Cadherin (3195, Cell Signaling Technology), vimentin (sc-6260, Santa Cruz Biotechnology), fibronectin (ab2413, Abcam, San Francisco, CA, USA), pH3, and CTGF (sc-14939, Santa Cruz Biotechnology), followed by Alexa Fluor–conjugated secondary antibody staining (Life Technologies, Carlsbad, CA, USA). Finally, cells or cryosections were mounted with mounting media containing propidium iodide or 4,6-diamidino-2-phenylindole (DAPI, Abcam), and visualized using the EVOS FL microscope (Thermo Fisher Scientific) or the Cytation 5 cell imaging system (BioTek Instruments, Winooski, VT, USA).

#### Western immunoblot analysis

Cells were lysed and mouse kidneys were homogenized in radioimmunoprecipitation assay buffer supplemented with the protease inhibitor cocktail (Thermo Fisher Scientific). Samples were subjected to Western immunoblot analysis as described before^8^. The blots were incubated with GSK3β, E-cadherin, vimentin, fibronectin (ab2413, Abcam; sc-9068, Santa Cruz Biotechnology), CTGF, PAI-1, pH3, HA (sc-7392, Santa Cruz Biotechnology), GAPDH (sc-32233, Santa Cruz Biotechnology), and β-actin (sc-81178, Santa Cruz Biotechnology). For immunoblot analysis, bands were scanned and the integrated pixel density was determined using the ImageJ analysis program, version 1.52a (National Institutes of Health, Bethesda, MD, USA).

#### Immunoprecipitation

Immunoprecipitation was carried out using an established method as described previously^13^. Briefly, nuclear fractions of cells or kidneys were prepared with the NE-PER kit (Thermo Fisher Scientific) according to the manufacturer’s instruction. Samples were incubated with anti-CREB-binding protein (CBP) antibody (sc-7300, Santa Cruz Biotechnology) and then precipitated by incubating with protein A/G-agarose overnight. The precipitated complexes were collected, washed, separated on SDS-polyacrylamide gels, and subjected to immunoblot analysis with anti-p-Smad2 (3108, Cell Signaling Technology), p-CREB (9198, Cell Signaling Technology), and CBP antibodies as indicated above.

#### Morphologic analysis of human kidney tissues

Human research participants were not specifically recruited for this study. Histology of the human kidney sections stained for GSK3β by peroxidase immunohistochemistry was obtained from previously published work^14,15^. The kidney tissues had been originally derived from archived excessive kidney biopsy tissues from patients with focal segmental glomerulosclerosis (FSGS) or pre-implant kidney biopsy specimens. The use of unidentified human biopsy specimens had conformed to the ethical guidelines of the 1975 Declaration of Helsinki.

#### Bioinformatics analysis

Renal tubulointerstitial transcriptome data of 9 normal kidney tissues from healthy living donors and 18 renal biopsy tissues from patients with FSGS were derived from European Renal cDNA Bank (ERCB) nephrotic syndrome study and collected in the Nephroseq database (www.nephroseq.org). Gene set enrichment analysis (GSEA) was performed based on GSE104954 to identify biological pathways that were associated with tubulointerstitial GSK3β and involved in CKD pathogenesis as previously described^16^. GSEA software was acquired from the Broad Institute (http://www.broad.mit.edu/gsea).

### Statistical analysis

All data are expressed as mean ± SD. All in vitro studies were repeated at least three times. Power analysis was performed to determine the adequate sample size of animal groups to measure changes in kidney function and renal injuries in a statistically significant manner. Software G*Power for sample size calculation was used as described before^17^. Statistical analysis of the data from multiple groups was performed by one-way ANOVA tests followed by Tukey’s tests. Data from two groups were compared by a two-sided Student’s *t*-test. Statistical analyses were performed using GraphPad Prism 7.0 software (GraphPad Software, San Diego, CA, USA) or SPSS 22 (IBM Corporation, Armonk, New York, USA). *P* < 0.05 was considered statistically significant.

## Results

### GSK3β is upregulated in renal tubules in progressive CKD and involved in renal fibrogenesis

**Fig. 1:**
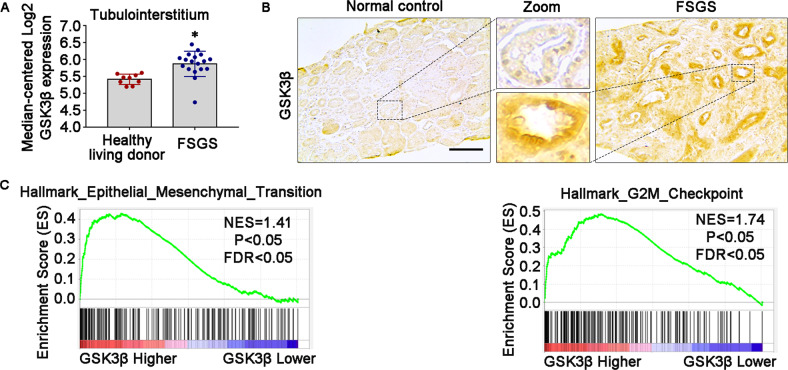
Renal tubular overexpression of GSK3β is involved in renal fibrogenesis in progressive CKD. **A** *Post hoc* bioinformatics analysis of renal tubulointerstitial transcriptome for the expression levels of GSK3β mRNA in kidney tubulointerstitial specimens procured from healthy living donors and patients with FSGS. Renal tubulointerstitial transcriptome data were derived from www.Nephroseq.org on the basis of the ERCB nephrotic syndrome datasets. *P < 0.05 versus healthy living donor group (*n* = 9–18). **B** Renal biopsy specimens from patients with FSGS and normal controls of pre-implant kidney biopsy specimens were processed for immunohistochemistry staining for GSK3β. Representative micrographs were shown. Scale bar = 100 μm. **C** Gene set enrichment analysis (GSEA) demonstrated that the predefined gene sets “Epithelial_Mesanchymal_Transition” and “G2M_Checkpoints” exhibited significant enrichment in high-expression of GSK3β versus low-expression of GSK3β in renal tubulointerstitial specimens procured from healthy living donors and patients with CKD based on data derived from GSE104954 dataset. ERCB, European Renal cDNA Bank; NES, normalized enrichment score; FDR, false discovery rate; FSGS, focal segmental glomerulosclerosis.

Recent studies have implicated GSK3β in diverse kidney diseases, including glomerular diseases^8^, diabetic nephropathy^18^, and acute kidney injury (AKI)^9^. However, its role in progressive CKD and renal fibrosis is unclear. To address this issue, renal tubulointerstitial expression of GSK3β in progressive CKD, as opposed to normal controls, was profiled by a post hoc bioinformatics analysis of the publicly available kidney transcriptome database Nephroseq^19^. As shown in Fig. 1A, based on a dataset from ERCB in the Nephroseq database, mRNA expression levels of GSK3β in kidney tubulointerstitial specimens procured from patients with FSGS were significantly higher than those in healthy living donors. The kidney tubulointerstitium is known to consist of heterogeneous cell types, including renal TECs, vascular cells, and interstitial cells. To locate the expression of GSK3β in tubulointerstitium, renal biopsy specimens from patients with FSGS and pre-implant kidney biopsy specimens were subjected to immunohistochemistry staining for GSK3β. In accordance with the above transcriptome data, immunostaining of GSK3β in renal tubulointerstitium was evidently more intense in FSGS patients as compared with normal controls. The upregulated expression of GSK3β was predominantly located in renal tubules (Fig. 1B).

Moreover, to determine whether GSK3β is involved in the kidney fibrogenic process, GSEA was performed based on the GSE104954 dataset (Tubulointerstitial transcriptome from ERCB subjects with CKD). As shown in Fig. 1C, the predefined hallmark gene sets implicated in fibrogenesis, including “Epithelial_Mesanchymal_Transition” and “G2M_Checkpoints”, exhibited significant enrichment in high-expression of GSK3β versus low-expression of GSK3β in kidney tubulointerstitial specimens procured from patients with CKD.

### Pharmacological inhibition of GSK3β attenuates the TGF-β1 elicited renal TEC profibrogenic plasticity

**Fig. 2:**
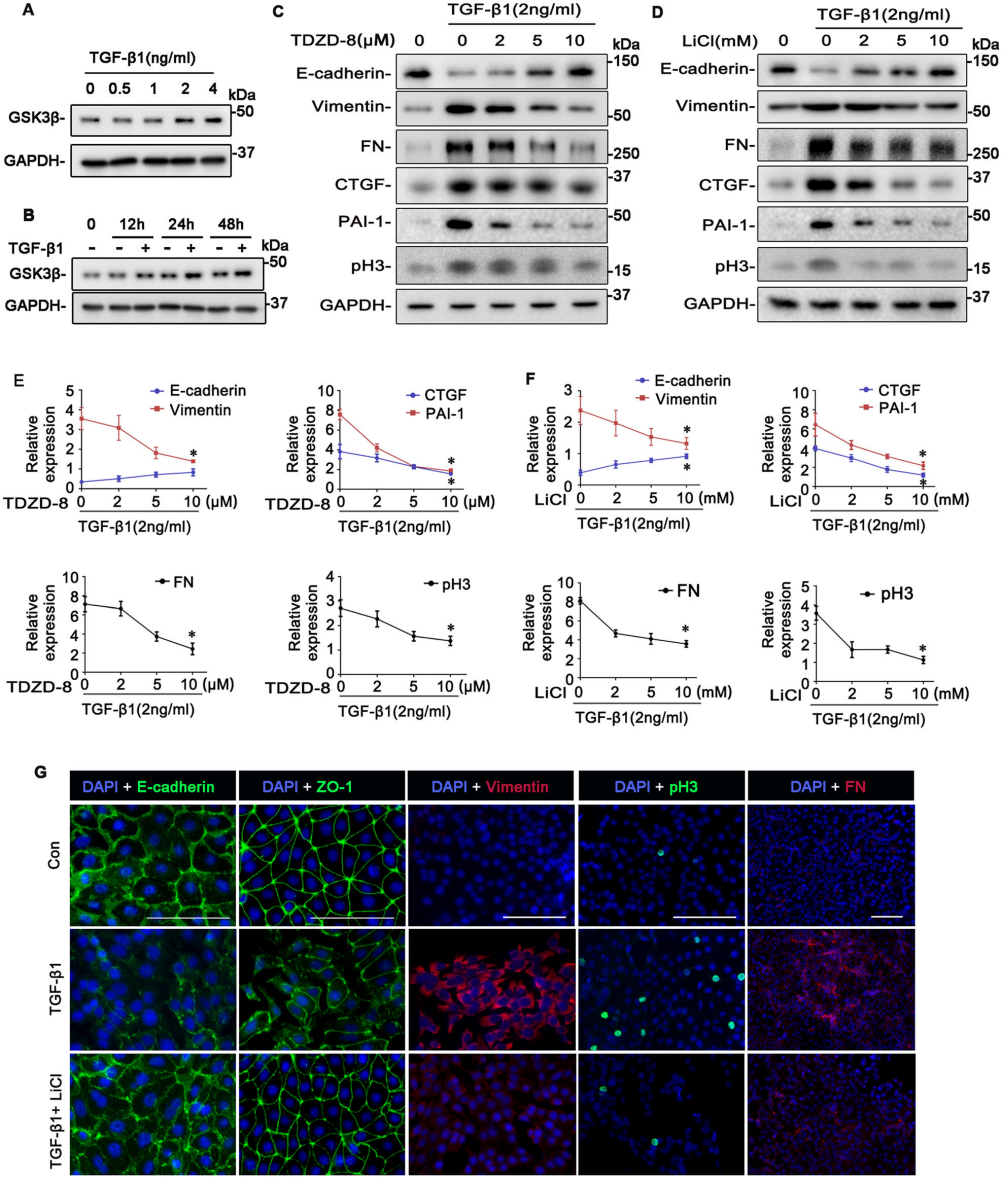
Pharmacological inhibition of GSK3β by TDZD-8 or LiCl attenuates the TGF-β1 induced TEC profibrogenic plasticity in a dose-dependent fashion. **A** The immortalized murine renal proximal tubular epithelial cells (TKPT) were treated with or without TGF-β1 (0.5, 1, 2, 4 ng/ml) for 24 h. Cell lysates were prepared for immunoblot analysis for GSK3β and GAPDH. Representative immunoblots were shown. **B** TKPT were treated with or without TGF-β1 (2 ng/ml) for 12 h, 24 h, or 48 h. Cell lysates were prepared for immunoblot analysis for GSK3β and GAPDH. Representative immunoblots were shown. **C** TKPT were treated with TGF-β1 (2 ng/ml) for 24 h following pretreatment with different dose of TDZD-8 (0, 2, 5, 10 μmol/L) or (**D**) LiCl (0, 2, 5, 10 mmol/L) for 30 min. Cell lysates were prepared for immunoblot analysis for E-cadherin, vimentin, fibronectin (FN), PAI-1, CTGF, pH3, and GAPDH. Representative immunoblots were shown. **E**, **F** Densitometric analyses of the expression of E-cadherin, vimentin, FN, PAI-1, CTGF, and pH3, as normalized to the expression of GAPDH based on immunoblot analysis. ^*^P < 0.05 versus TGF-β1 treatment group (*n* = 3, *t*-test). **G** TKPT were treated with TGF-β1 in the presence or absence of LiCl (10 mmol/L) for 24 h and then fixed for immunofluorescent staining of E-cadherin, ZO-1, vimentin, pH3, and FN with nuclear counterstaining with DAPI. Representative fluorescent micrographs were shown. Scale bar = 100 μm. CTGF, connective tissue growth factor; DAPI, 4′,6-diamidino-2-phenylindole; FN, fibronectin; GAPDH, glyceraldehyde-3-phosphate dehydrogenase; PAI-1, plasminogen activator inhibition-1; pH3, phosphorylated histone H3; ZO-1, zonula occludens-1.

**Fig. 3:**
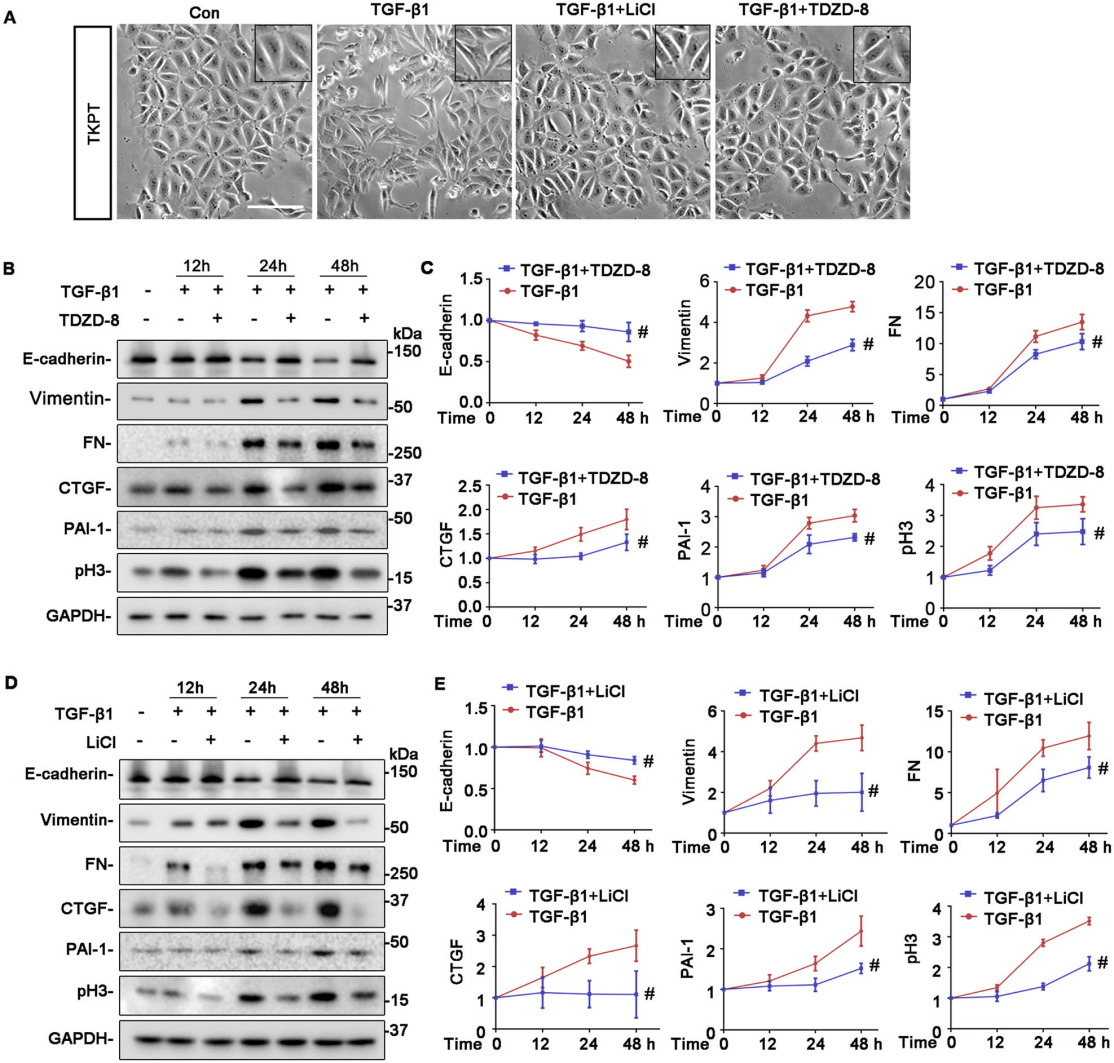
The time course of the effects of TDZD-8 or LiCl on the TGF-β1 induced TEC profibrogenic plasticity. **A** TKPT were treated with TGF-β1 (2 ng/ml) for 24 h following pretreatment with TDZD-8 (10 μmol/L) or LiCl (10 mmol/L) for 30 min. Representative phase-contrast micrographs were shown. Scale bar = 100 μm. **B** TKPT were treated with TGF-β1(2 ng/ml) for 12 h, 24 h, 48 h following pretreatment with TDZD-8 (10 μmol/L) or (**D**) LiCl (10 mmol/L) for 30 min. Cell lysates were prepared for immunoblot analysis for E-cadherin, vimentin, FN, PAI-1, CTGF, pH3, and GAPDH. Representative immunoblots were shown. **C**, **E** Densitometric analyses of the expression of E-cadherin, vimentin, FN, PAI-1, CTGF, and pH3, as normalized to the expression of GAPDH and expressed as fold changes relative to the control group based on immunoblot analysis. ^#^*P* < 0.05 versus TGF-β1  group (*n* = 3, *t*-test).

To further decipher the role of renal tubule-specific GSK3β in renal fibrogenesis, we employed an in vitro model of tubulointerstitial fibrosis, in which cultured murine TECs were treated with TGF-β1, a prototype of profibrotic cytokines centrally implicated in renal fibrosis^20,21^. In agreement with the increased tubular expression of GSK3β in patients with progressive CKD, TGF-β1 treatment induced the TEC expression of GSK3β in a dose and time-dependent fashion (Fig. 2A, B). This was associated with evident molecular changes of TEC profibrogenic plasticity, including loss of epithelial markers like E-cadherin adhesive junctions and ZO-1 tight junctions, acquisition of mesenchymal phenotypes like vimentin intermediate filaments, increased expression of fibrous ECM components like fibronectin, cell cycle arrest at the G2/M phase marked by de novo expression of pH3, and overproduction of profibrotic cytokines like CTGF and PAI-1, as measured by immunoblot analysis of cell lysates in combination with densitometry or by fluorescent immunocytochemistry staining of fixed cells (Fig. 2C–G). In parallel with the molecular changes, TECs underwent morphologic changes from the cobblestone-like cuboidal appearance of typical epithelial cells to a dispersed fusiform shape of mesenchymal cells (Fig. 3A). In contrast, blockade of GSK3β by the highly selective non-ATP competitive small molecule inhibitor TDZD-8, or by the classical inhibitor lithium salt, mitigated the TGF-β1-induced molecular and morphologic changes of TEC profibrogenic plasticity in a dose and time-dependent manner (Figs. 2 and 3).

### GSK3β regulates TGF-β1-elicited TEC profibrogenic plasticity

**Fig. 4:**
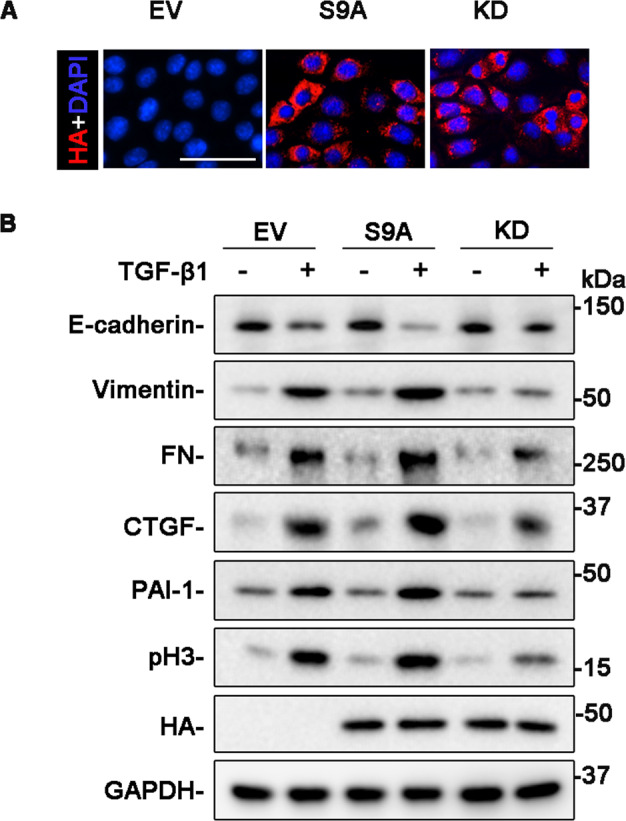
The permissive effect of GSK3β on the TGF-β1 induced TEC profibrogenic plasticity. TKPT were subjected to liposome-mediated transiently transfection with vectors encoding the empty vector (EV), dominant-negative (KD), or constitutively active (S9A) GSK3β. **A** Representative micrographs of fluorescent immunocytochemistry staining for HA (red) with nuclear counterstaining with DAPI (blue). Scale bar = 50 μm. **B** After transfection, cells were treated with 2 ng/ml of TGF-β1 for 24 h. Cell lysates were collected and subjected to immunoblot analysis for E-cadherin, vimentin, FN, PAI-1, CTGF, pH3, HA, and GAPDH. Representative immunoblots were shown.

To determine if there is a direct causal relationship between GSK3β and TGF-β1-induced TEC profibrogenic plasticity, the activity of GSK3β in cultured TECs was manipulated by forced expression of vectors encoding the HA-conjugated KD, or S9A with a transfection efficiency of more than 80%, as estimated by immunostaining (Fig. 4A) and immunoblot analysis (Fig. 4B) for HA. After TGF-β1 exposure, ectopic expression of the S9A mutant of GSK3β sensitized cell dedifferentiation, as evidenced by more reduction in E-cadherin expression and more induction of vimentin expression. Meanwhile, the potentiated production of fibronectin, PAI-1, and CTGF was also noted in S9A-expressing cells. Besides, cell cycle arrest at the G2/M phase, shown by expression of pH3, was also amplified by forced expression of S9A (Fig. 4B). Conversely, all these molecular changes of TEC profibrogenic plasticity elicited by TGF-β1 were blunted in KD-expressing cells (Fig. 4B), reminiscent of the effect of lithium or TDZD-8, entailing that GSK3β plays a permissive role in TGF-β1-induced TEC profibrogenic plasticity.

### GSK3β regulates TGF-β1 signaling in TECs via a CREB dependent mechanism

**Fig. 5:**
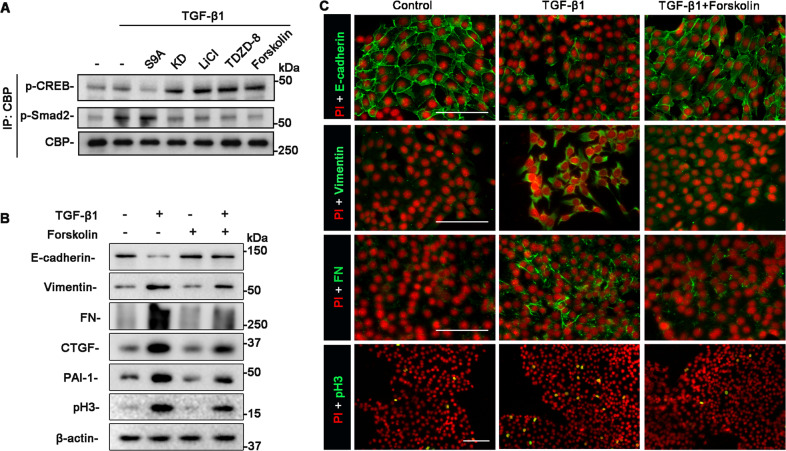
GSK3β suppresses the activity of CREB, which competes with Smad proteins for binding to CBP and abolishes the TGF-β1 induced TEC profibrogenic plasticity. TKPT cells were pretreated with lithium, TDZD-8, or forskolin, or were subjected to liposome-mediated transient transfection with vectors encoding KD, or S9A, followed by TGF-β1 treatment for 6 h. **A** Cell lysates were subjected to immunoprecipitation with the anti-CBP antibody. Immunoprecipitates were processed for immunoblot analysis for p-CREB (ser 133), p-Smad2, and CBP. Representative immunoblots were shown. **B** Cell lysates prepared from TKPT cells with indicated treatments were processed for immunoblot analysis for E-cadherin, vimentin, FN, CTGF, PAI-1, pH3, and β-actin. Representative immunoblots were shown. **C** TKPT were fixed after indicated treatments and prepared for immunofluorescent staining for E-cadherin, vimentin, FN, and pH3 with nuclear counterstaining with PI. Representative fluorescent micrographs were shown. Scale Bar = 100 μm. CREB, cAMP response element-binding protein; CBP, CREB-binding protein; PI, propidium iodide.

Full activation of the TGF-β1/Smad signaling pathway requires the binding of Smad proteins to several essential transcriptional coactivators, including the CBP^22^. Meanwhile, CREB also interacts with CBP and thus is able to compete for binding to CBP. Previous studies have shown that GSK3β regulates CREB signaling pathway in various cells^23–25^. This prompted us to determine if GSK3β also dictates the activity of CREB in TECs and if this action affects the TGF-β1/Smad signaling pathway. To this end, lithium or TDZD-8-treated TECs and S9A or KD-expressing TECs were injured with TGF-β1. Cell lysates were subsequently subjected to immunoprecipitation with the anti-CBP antibody. Shown by immunoblot analysis of immunoprecipitates in Fig. 5A, inhibition of GSK3β activity by lithium or TDZD-8, or ectopic expression of KD augmented the amount of activated CREB (phosphorylated on serine 133) that coprecipitated with CBP, denoting a promotional effect of GSK3β inhibition on the activity of CREB and CBP recruitment to CREB. This was reciprocally associated with diminished binding of CBP to p-Smad2, suggestive of repressed TGF-β1/Smad signaling activity. Forskolin, a specific activator of adenylyl cyclase that raises levels of cAMP and triggers the cAMP-CREB signaling pathway, mimicked the effects of GSK3β inhibition and substantially abrogated the TGF-β1 elicited molecular changes of TEC profibrogenic plasticity, as shown by immunoblot analysis and immunofluorescence staining (Fig. 5B–C), entailing that increased CREB activity is sufficient to mitigate the TGF-β1/Smad signaling in TECs and the consequent maladaptive plasticity. Conversely, increasing GSK3β activity by forced expression of S9A suppressed CBP binding to CREB but facilitated CBP recruitment to p-Smad2 (Fig. 5A), consistent with a reinforced TGF-β1/Smad signaling activity. Taken together, the above findings suggest that GSK3β modulates the competition between CREB and Smad proteins for binding to the shared transcriptional coactivator CBP and that GSK3β inhibition offsets the TGF-β1/Smad signaling activity that drives molecular changes of TEC profibrogenic plasticity in progressive CKD.

### Targeting of GSK3β in TEC improves kidney fibrosis in mice with FA nephropathy

**Fig. 6:**
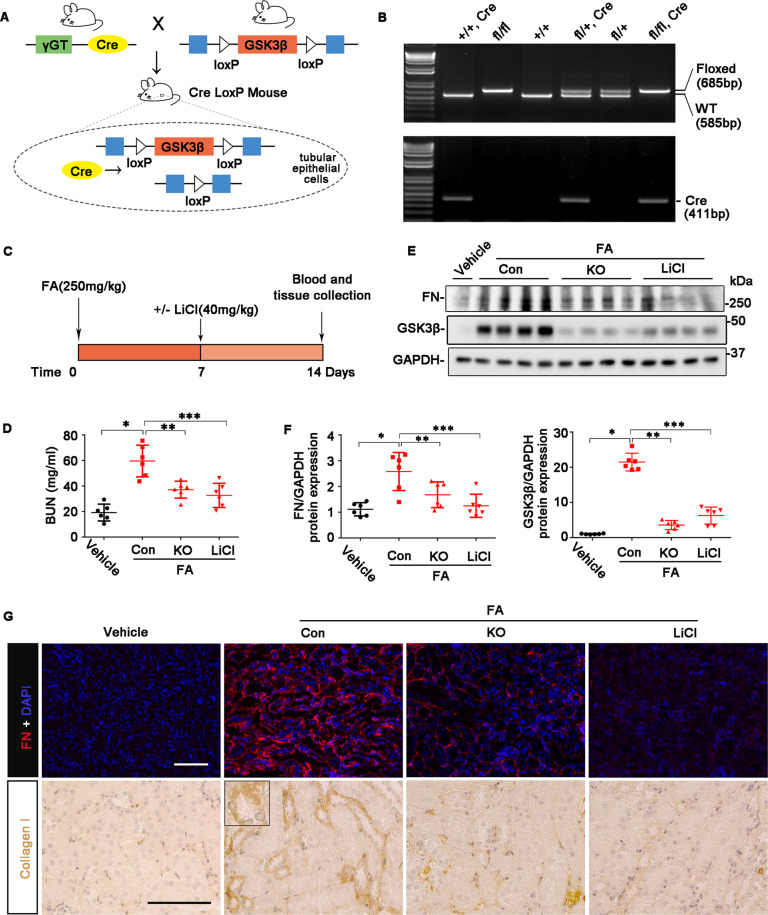
Renal TEC-specific GSK3β gene deletion or lithium improves kidney fibrosis in folic FA-elicited progressive CKD. **A** Schematic diagram of the strategy for generation of mice with renal tubular epithelial cell-specific GSK3β gene knockout (KO). **B** Representative photos showing PCR analysis of the genomic DNA extracted from the clipped tail tissues. Genotypes of representative litters are indicated; fl, GSK3β floxed; WT, wild type. Mice with the genotype GSK3β^*fl/fl*^, Cre were used as KO group. Control littermates served as control (Con). **C** The schematic diagram depicts the experimental design. Mice were injured with an injection of FA (250 mg/kg) and 7 days later were treated with or without LiCl (40 mg/kg). Mice were followed up and euthanized on day 14 after FA injection, blood and kidney specimens were collected and processed for further examinations. **D** Quantification of blood urea nitrogen (BUN). *^,^ **^,^ ***P < 0.05 (*n* = 6, ANOVA followed by Tukey’s test). **E** Kidney specimens were homogenized for immunoblot analysis for FN, GSK3β, and GAPDH. Representative immunoblots were shown. **F** Densitometric analyses of the expression of FN and GSK3β, as normalized to the expression of GAPDH and expressed as fold changes relative to the control group based on immunoblot analysis. *^,^ **^,^ ***P < 0.05 (*n* = 6, ANOVA followed by Tukey’s test). **G** Representative micrographs of immunofluorescence staining for FN (red) with nuclear counterstaining with DAPI (blue), or immunohistochemistry staining for collagen I. Scale bar = 100 μm.

To validate the role of GSK3β in TEC profibrogenic plasticity and kidney fibrosis in vivo, we employed the TEC-specific GSK3β KO mice (Fig. 6A), which were generated based on the Cre-loxP recombination technology by crossing GSK3β-floxed mice with γGT. Cre transgenic mice, as confirmed by tail-snip genotyping (Fig. 6B). KO mice together with the control littermates were insulted by a single injection of FA (250 mg/kg). On day 7 after FA injection, mice received a microdose of lithium (40 mg/kg) or an equal molar amount of sodium chloride-based on our previous experience^9^ and were followed up for another 7 days (Fig. 6C). FA injury elicited evident kidney dysfunction in control mice, marked by significant increases in serum BUN levels (Fig. 6D). In consistency, as shown by immunoblot analysis of kidney homogenates and by immunohistochemistry staining, renal expression of fibrous ECM components like fibronectin or collagen I was drastically increased, with the majority being located to renal tubulointerstitium (Fig. 6E–G), indicative of progressive CKD and kidney fibrosis. This coincided with GSK3β overexpression, denoting GSK3β hyperactivity. As expected, ablation of GSK3β in TECs or LiCl treatment successfully mitigated renal overexpression of GSK3β in FA-injured mice (Fig. 6E, F). This was associated with correction of renal dysfunction, as well as improvement in kidney fibrosis, as revealed by the lessened expression of fibronectin and collagen I. As a ubiquitously expressed kinase, GSK3β is also expressed in other kidney cells, such as fibroblasts, vascular endothelial cells, and inflammatory cells. It is plausible that GSK3β in other kidney cells may likewise play a role in kidney fibrosis, and is targetable by systemic lithium treatment but not by renal tubules-restricted GSK3β inhibition in KO mice. Indeed, as shown by immunoblot analysis and immunostaining for fibronectin or collagen I, lithium-treated mice had a tendency to achieve a greater anti-fibrotic efficacy than the KO mice.

### GSK3β facilitates TEC profibrogenic plasticity in FA-elicited progressive CKD

**Fig. 7:**
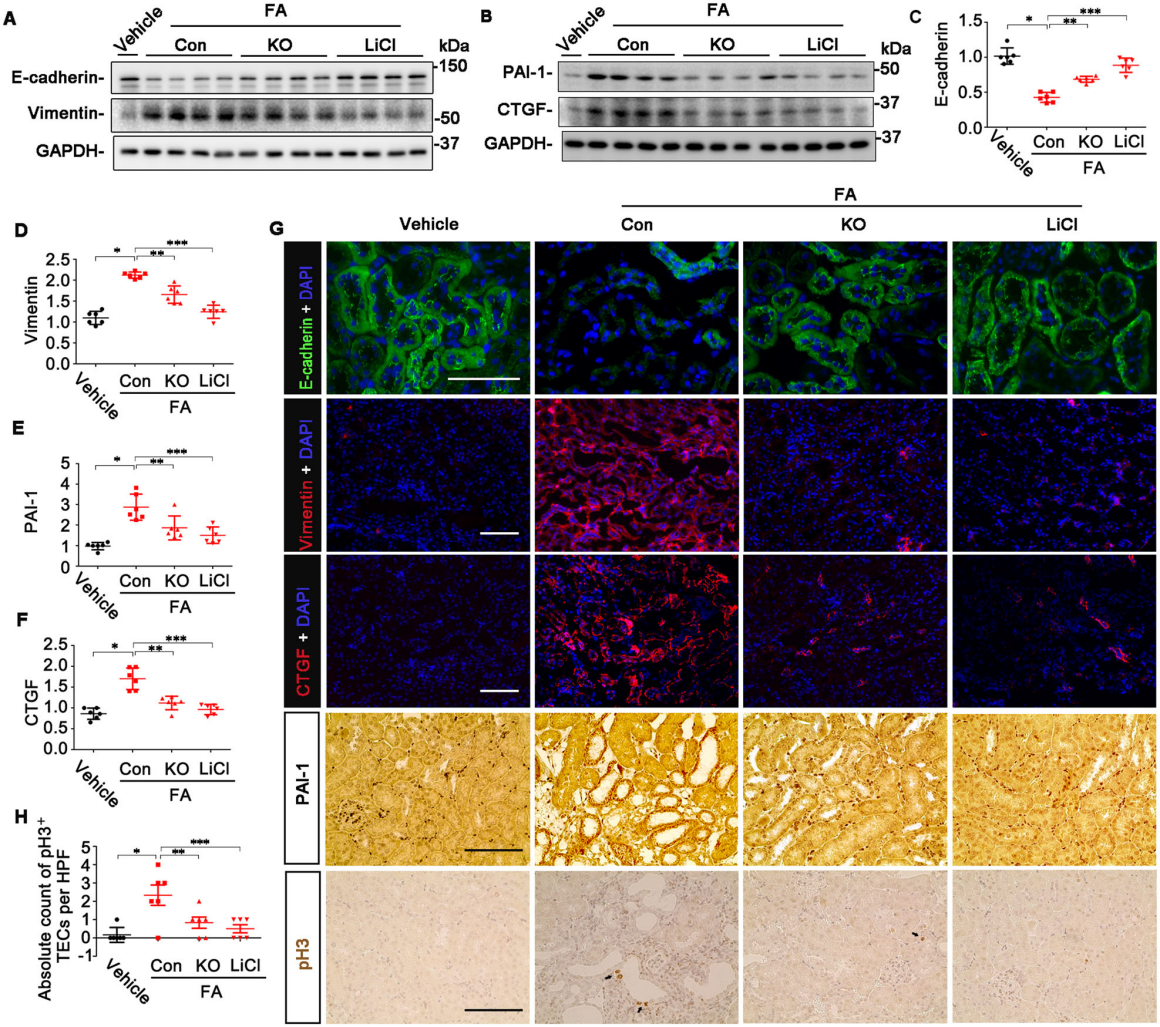
Profibrogenic plasticity of renal tubules in the mouse model of FA nephropathy is abrogated by TEC-specific GSK3β ablation or by lithium. **A**, **B** Kidney homogenates were processed for immunoblot analysis for E-cadherin, vimentin, PAI-1, CTGF, and GAPDH. Representative immunoblots were shown. **C**–**F** Densitometric analyses of the expression levels of E-cadherin, vimentin, PAI-1, and CTGF, as normalized to the GAPDH expression and expressed as fold changes relative to the control group. *P, **P, ***P < 0.05 (*n* = 6, ANOVA followed by Tukey’s test). **G** Representative micrographs of immunofluorescence staining for E-cadherin (green), vimentin (red), and CTGF (red) with nuclear counterstaining with DAPI (blue), or peroxidase immunohistochemistry staining for PAI-1 and pH3 in kidney tissues. Scale bar = 100 μm. **H** Absolute counting of the number of TEC cells positive for pH3 staining per high power field (HPF). *P, **P, ***P < 0.05 (*n* = 6, ANOVA followed by Tukey’s test).

Burgeoning evidence suggests that renal tubules undergo profibrogenic plasticity in progressive CKD and thereby play a key role in driving the development and progression of kidney fibrosis^26^. To assess renal tubule profibrogenic plasticity in the FA-injured mice, kidney specimens were processed for immunoblot analysis followed by densitometry. As shown in Fig. 7A–F, renal expression of E-cadherin, a renal TEC marker, plummeted in FA-injured control mice, in parallel with a substantial induction of vimentin intermediate filaments, a marker of mesenchymal phenotypes, and overproduction of profibrotic cytokines like PAI-1 and CTGF. To locate these molecular changes in the kidney, immunohistochemistry staining of kidney specimens was performed and demonstrated that loss of E-cadherin and increased expressions of vimentin, PAI-1, and CTGF were mainly localized to renal tubules (Fig. 7G), entailing renal tubular dedifferentiation and acquisition of profibrogenic phenotypes. This was concomitant with evident renal TEC cell cycle arrest at the G2/M phase, as probed by immunostaining for pH3 (Fig. 7G, H). All these molecular changes of renal tubular profibrogenic plasticity upon FA injury were markedly abrogated by lithium treatment or by GSK3β ablation in KO mice.

### GSK3β inhibition promotes CREB activity in renal tubules in FA-elicited progressive CKD

**Fig. 8:**
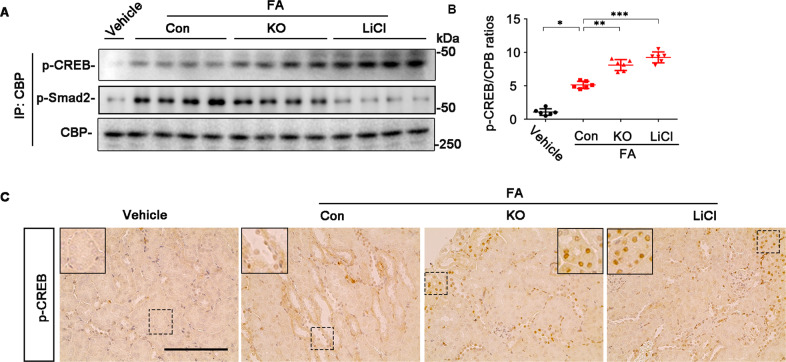
GSK3β inhibition in mice with FA nephropathy increases CREB activity in renal tubules and favors CREB competition with Smad for binding to CBP. **A** Kidney homogenates were subjected to immunoprecipitation with the anti-CBP antibody and immunoprecipitates were processed for immunoblot analysis for indicated molecules. Representative immunoblots were shown. **B** Densitometric analysis of the levels of p-CREB that co-precipitated with CBP, as normalized to the CBP levels and expressed as fold changes relative to the control group. *P, **P, ***P < 0.01 (*n* = 6, ANOVA followed by Tukey’s tests). **C** Representative micrographs of immunohistochemistry staining for p-CREB. Scale bar = 100 μm.

To ascertain if GSK3β inhibition-improved renal tubular profibrogenic plasticity in FA nephropathy is associated with a change in the activity of CREB as posited by the above in vitro studies, kidney homogenates were processed for immunoprecipitation with anti-CBP antibody followed by immunoblot analysis. As shown in Fig. 8A, B, GSK3β inhibition by lithium treatment or by GSK3β ablation in KO mice considerably increased the amount of activated CREB that coprecipitated with CBP, denoting a promoted binding of CBP to CREB. This was reciprocally associated with reduced coprecipitation of p-Smad2 that with CBP, suggesting mitigated TGF-β1/Smad signaling. Immunohistochemistry staining indicated that the increased CREB activity occurred mostly in renal tubules in KO or lithium-treated mice (Fig. 8C).

## Discussion

Renal tubulointerstitial fibrosis is the final common pathway for various CKDs that drives the progression to ESRD. The present work showed that GSK3β expression was amplified in renal tubular cells in patients with progressive CKD, as well as in both in vitro and in vivo models of renal fibrosis, concomitant with renal TEC fibrogenic plasticity. Moreover, targeting of GSK3β in renal tubules via genetic knockout or by lithium, a standard inhibitor of GSK3β and FDA-approved mood stabilizer, effectively preserved renal TEC phenotypes and ameliorated renal fibrosis in mice with FA nephropathy. To the best of our knowledge, this study is the first to demonstrate that renal tubule-specific GSK3β is involved in renal TEC fibrogenic plasticity and mediates renal fibrogenesis.

As a highly conserved serine/threonine kinase initially discovered to mediate the insulin signaling pathway and glycogen biosynthesis, GSK3β has also been implicated in many other pathophysiologic processes and conditions, including organ injury and repair, carcinogenesis, neurodegenerative diseases, and more recently kidney diseases^27^. In the kidney, GSK3β is mainly expressed in glomeruli and proximal renal tubules^14^. Upon acute injuries, the activity of GSK3β in glomeruli or renal tubules is augmented secondary to the repressed inhibitory phosphorylation. This GSK3β hyperactivity has been shown to aggravate kidney cell death and acute renal injury via multiple mechanisms, including sensitization of mitochondria permeability transition, disruption of cytoskeleton integrity, and potentiation of NF-κB-dependent inflammatory responses^28–30^. In complementary studies, therapeutic targeting of GSK3β was able to protect against kidney dysfunction and mitigate acute renal injury in animal models of acute glomerulopathy^8^ or AKI^9^, and improve the subsequent AKI to CKD transition^31^. Nevertheless, despite an unequivocal role of GSK3β in acute kidney diseases, it is not fully understood if GSK3β contributes to chronic renal fibrogenesis and progressive CKD. Of note, the process of kidney fibrosis implicates multifarious kidney cells, including renal TECs, interstitial fibroblasts, vascular endothelial cells, and inflammatory cells. Among these, renal TECs are not only the target or victim of kidney injury but also a *sine qua non* of renal fibrotic changes. In progressive CKD, the persistent injury may cause TEC dedifferentiation, cell cycle arrest, and mesenchymal phenotypic switch, ultimately leading to excessive accumulation of fibrous ECM and kidney scarring^26^. Akin to the findings in the scenario of AKI, the activity of GSK3β in renal TECs is likewise augmented, marked by GSK3β overexpression, in diverse CKDs, such as diabetic nephropathy^18^, chronic allograft nephropathy^15,32^, and, in this study, FSGS and FA nephropathy. Our data indicated that this increased GSK3β activity may confer a permissive effect on TEC fibrogenic plasticity and contribute to renal fibrosis. Consistent with our findings, a number of studies also demonstrated that GSK3β exerts a pro-fibrotic effect in many other organ systems. For instance, inhibition of GSK3β by 9ING41, a highly selective small-molecule inhibitor, was able to improve bleomycin-induced pulmonary fibrosis in mice^33^. In addition, morin, a dietary flavonoid, was able to reduce GSK3β expression in hepatic stellate cells and thereby meliorates diethylnitrosamine-induced liver fibrosis in rats^34^. Moreover, inhibition of GSK3β activity via cardiac-specific overexpression of dominant-negative GSK3β resulted in better left ventricular function and less fibrosis and apoptosis in mice subjected to transverse aortic constriction^35^.

TGF-β1 signaling is one of the master signaling pathways in driving the development and progression of renal fibrosis. It acts on diverse renal parenchymal cells to induce cellular dedifferentiation, transdifferentiation, migration, and overproduction of fibrous ECM and profibrotic cytokines, such as PAI-1 and CTGF^36^. Most studies have focused on the effect of TGF-β1 on fibroblast-like cells. However, there is data demonstrating that specific knockout of TGFBR2 in matrix-producing interstitial cells minimally diminished overall renal fibrosis in mice with unilateral ureteric obstruction (UUO) or aristolochic acid nephropathy^37^. This finding suggests that the effect of TGF-β1 on non-fibroblast-like cells is also highly involved in the development of renal fibrosis. Indeed, Meng et al. selectively ablated TGFBR2 in renal TECs and showed a significant protective effect on UUO-induced renal fibrosis^38^. In renal TECs, TGF-β1 acts through the TGFBR/Smad signaling to trigger molecular changes of profibrogenic plasticity. Then, how does GSK3β regulate the TGF-β1/Smad signaling? Although the exact mechanism is still elusive, the following possibilities are in accordance with our findings and previous reports. It is likely that GSK3β is able to modulate the competition between Smad and CREB for binding to the shared transcriptional coactivator CBP, which is crucial for the full activation of TGF-β1/Smad signaling to trigger the TEC profibrogenic plasticity. In support of this, it has been well established that GSK3β is capable of modulating the activity of CREB in multiple cells, including immune cells, neurocytes, and fibroblasts^39,40^. In agreement, the present study showed that binding of activated CREB to CBP in TGF-β1-treated renal TECs was enhanced after GSK3β inhibition but blunted by ectopic expression of S9A. How GSK3β regulates the activity of CREB is still poorly understood. It is known that phosphorylation of CREB at serine 133 is required for CBP recruitment, and the ensuing DNA binding and transcription^41^. However, the cognate phosphorylation site in CREB for GSK3β is not serine 133 but serine 129 as predicted by sequence analysis (data not shown) and validated by a number of studies^39,42^. In accordance, phosphorylation of CREB at serine 133 creates the consensus sequence motif, SXXXS(P), thus priming CREB as a substrate for hierarchical phosphorylation of serine-129 by GSK3β^39,43^. It seems that phosphorylation of CREB by protein kinase A increased the DNA binding of CREB, whereas secondary phosphorylation of primed CREB by GSK3β attenuated protein kinase A stimulation of CREB DNA binding activity, implying that phosphorylation by GSK3β at serine 129 acts as a suppressive signal for CREB activity^39,42^. This effect was likely attributable to changes in the conformational structure and net charges of CREB after GSK3β-mediated phosphorylation^42^. This inhibitory effect of GSK3β on CREB activity has been reproducibly demonstrated in SH-SY5Y cells^39^, fibroblast cells^23^, and here again in renal TECs.

In summary, this study highlights a permissive effect of GSK3β on profibrogenic plasticity of renal TECs and renal fibrogenesis in progressive CKD. GSK3β modulates the competition between CREB signaling and TGF-β1/Smad signaling for the recruitment of the shared transcriptional coactivator CBP. GSK3β inhibition intercepts the TGF-β1/Smad signaling activity that drives molecular changes of TEC profibrogenic plasticity and ameliorates renal fibrosis in CKD (Supplementary Fig. 1). Our findings suggest that therapeutic targeting of GSK3β is likely a pragmatic approach to avert the maladaptive plasticity of renal TEC in progressive CKD and mitigate renal fibrosis.

## Supplementary information

Supplementary Figure

Supplementary Figure legend
