# Supplementary figures and images for: Permissive effect of GSK3β on profibrogenic plasticity of renal tubular cells in progressive chronic kidney disease
Source: Cell Death Dis. 2021 Apr 30;12(5):432. doi: 10.1038/s41419-021-03709-5 (PMC8087712; doi:10.1038/s41419-021-03709-5)

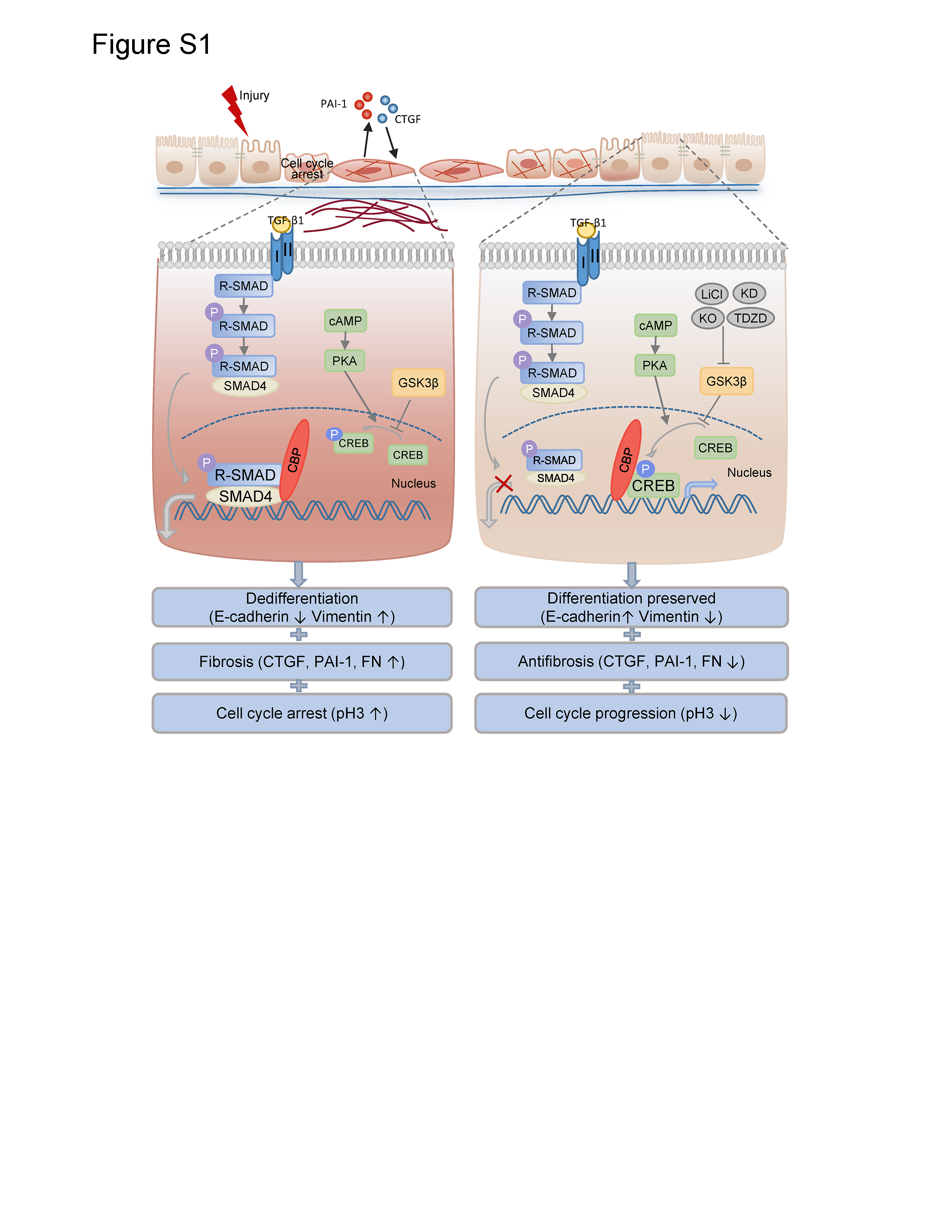

Supplement: Supplementary file 1 — Supplementary Figure [file 41419_2021_3709_MOESM1_ESM.tif]
