# Supplementary material for: Permissive effect of GSK3β on profibrogenic plasticity of renal tubular cells in progressive chronic kidney disease
Source: Cell Death Dis. 2021 Apr 30;12(5):432. doi: 10.1038/s41419-021-03709-5 (PMC8087712; doi:10.1038/s41419-021-03709-5)
Supplement: Supplementary file 2 — Supplementary Figure legend [file 41419_2021_3709_MOESM2_ESM.docx]

**Supplementary Figure legend**

**Figure S1. *Schematic depicting the role of GSK3β in regulating renal TEC profibrogenic plasticity in progressive CKD.*** The CREB signaling is able to compete with the TGF-β1/Smad signaling for recruiting the shared transcriptional coactivator CBP, which is essential for TGF-β1/Smad signaling pathway to drive molecular changes of renal TEC profibrogenic plasticity, including dedifferentiation and transdifferentiation, characterized respectively by loss of epithelial E-cadherin and acquisition of mesenchymal phenotypes like vimentin intermediate filaments, overproduction of fibrous ECM components like fibronectin and profibrotic cytokines like PAI-1 and CTGF, and cell cycle arrest at the G2/M phase marked by de novo expression of pH3 at serine 10. GSK3β regulates the activity of CREB and thereby affects the competition between CREB and Smad for binding to CBP. This study showed that GSK3β is overexpressed and hence hyperactive in renal tubules in progressive CKD and suppresses CREB activity, thus facilitating the TGF-β1/Smad signaling and the consequent renal TEC maladaptive plasticity. Conversely, targeting of GSK3β in renal TECs via genetic knockout or by lithium increases the activity of CREB and favors its competition for CBP, resulting in an intercepted TGF-β1/Smad signaling and an averted renal TEC profibrogenic plasticity.
